# Supplementary material for: Exploring Physician and Patient Perspectives on Expectations and Role Models Towards Chronic Pain Treatment in General Practice: A Qualitative Cross-Sectional Study
Source: Healthcare (Basel). 2025 Jan 18;13(2):187. doi: 10.3390/healthcare13020187 (PMC11764803; doi:10.3390/healthcare13020187)
Supplement: Supplementary file 1 [file healthcare-13-00187-s001.zip › healthcare-3398424-supplementary.pdf]

Supplementary Materials: Topics covered during data collection

Interview - General practitioner

- Socio-demographic characteristics of participants
- Treatment approach
- Assessment and monitoring
- Knowledge transfer/patient education
- Non-pharmacological interventions
- Rational pharmacotherapy
- Familiarity with relevant guidelines
- Prescribing of opioids
- Potential elements in a case management program
- What else is relevant?

Interview - Patient

- Pain history and experiences with treatment approaches
- Pain treatment in general practice
- Knowledge transfer/patient education
- Psychological aspects
- Use of analgesics and over the counter products
- Use of non-pharmacological interventions
- Social support
- Thought experiment
- Socio-demographic characteristics of participants
